# The Role of Occupational Therapy in Secondary Prevention of Diabetes

**DOI:** 10.1155/2019/3424727

**Published:** 2019-07-24

**Authors:** Xizi Shen, Xingping Shen

**Affiliations:** ^1^Schools of Medicine, The University of Queensland, Brisbane, Queensland, Australia; ^2^Department of Endocrinology, Zhongshan Hospital Xiamen University, Xiamen, Fujian, China

## Abstract

Diabetes mellitus is becoming a global health concern due to its prevalence and projected growth. Despite a growing number of interventions for secondary prevention of diabetes, there is a persistent poor glycemic control and poor adherence to the prescribed diabetes management regimen. In light of the tremendous costs of diabetes to both individuals and the society, it is pressing to find effective ways to improve diabetes self-management (DSM) and treatment adherence. Occupational therapists can bring values to the diabetes care team by evaluating multiple levels of influence on DSM, addressing personal and environmental barriers to well-being and DSM, and supporting patients to develop of a highly complex competences and skills to satisfactorily self-manage diabetes. This article summarizes two evidence-based, well-structured occupational therapy (OT) programs that use activity-based treatments and psychosocial strategies, respectively, to improve DSM abilities and to enhance quality of life. As the needs of adolescents with diabetes are quite different from other diabetic populations, this article also provides a summary of pediatric OT interventions that aim to facilitate autonomy and development of DSM ability among adolescents with diabetes. Evidence indicates that OT interventions can improve the quality of life and treatment adherence in patients with diabetes and hence should be continued and built on to address the increasing needs of diabetic populations.

## 1. Background

Diabetes mellitus is a growing health concern around the world. In 2016, 415 million people were afflicted with diabetes worldwide, and this number is expected to increase to 642 million, or one in every ten adults by the year 2040[1]. Diabetes, as a complex chronic disease, requires continuous medical care with multifactorial, risk-reducing approaches beyond glycemic control [2]. Therefore, ongoing patient self-management education and support are critical to preventing acute complications and reducing the risk of long-term complications [2]. The costs of diabetes are tremendous to both individuals and the society. Poor management of diabetes may give rise to an array of secondary complications with devastating consequences, including microvascular (such as retinopathy, neuropathy, and nephropathy) and macrovascular complications (such as myocardial infarction, angina pectoris, and stroke) [3], which adversely affect the well-being of individuals living with diabetes [4]. Globally, the costs of diabetes are expected to increase substantially from 1.3 trillion to 2.2 trillion U.S dollars in the baseline by 2030, which is equivalent to an increase in costs as a share of global GDP from 1.8% to 2.2% [5]. It is therefore pressing that effective ways of managing diabetes are found to reduce secondary complications and reduce the economic burden.

## 2. Diabetes Self-Management (DSM) Is Challenging

Landmark studies have clearly demonstrated that intensive glycemic control can significantly reduce the risk of developing microvascular and macrovascular complications in diabetes [6–8]. Since the risk of developing diabetes-related complications increases when values of glycated hemoglobin (HbA1c) are in excess of 6.5%, an HbA1c of less than 7% is generally a target goal for diabetes management [9, 10]. Given the nature of diabetes being a chronic disease, the potential to achieve the optimum glycemic control (HbA1c<7%) roots in patients' ability to consistently carry out self-management activities according to the prescribed diabetes management regimen [11].

Diabetes self-management (DSM) refers to that patients take on responsibility for nutrition, physical activities, insulin therapy, and glucose monitoring, to maintain a good metabolic control and reduce diabetes complications [12, 13]. DSM is not a single behavior but rather a complex, dynamic constellation of behaviors influenced by changes in social, environmental, and individual circumstances across the lifespan [14]. Patients are required to take an active role in restructuring daily habits and routines to take medications, monitor blood glucose, follow a diet, and exercise regularly [6, 7]. Such massive behavioral changes for anyone could be difficult. Firstly, patients' psychosocial well-being is particularly challenged when they are confronted with the burden of changing their current self-care regimen, especially among older adults who have to modify their longstanding habits acquired during their lifetime [15]. Secondly, taking diabetes medications, following a diet, and constantly monitoring glucose levels are difficult activities that necessitate ongoing motivation and gradual readiness [16]. Without these abilities, many patients, children and young adults in particular, have found DSM activities burdensome and challenging to persist [17, 18]. Thirdly, there are limited resources to support health professionals in addressing patients' psychosocial needs [17, 19]. Many health professionals reported feeling uncomfortable and unconfident in discussing diabetes-related psychosocial concerns [19]. It is, therefore, not surprising that many patients are experiencing what Rubin called “Diabetes overwhelms” which refers to patients' reacting to prescribed self-care changes with feelings of distress or burnout [20].

As a result of the aforementioned reasons, poor DSM is common among patients despite our growing understanding of diabetes. Researchers reported that in 2002 less than 50% of individuals receiving diabetes education had carried out self-management activities successfully in accordance with their diabetes management regimen, and only around 50% of patients had achieved the optimum glycemic control (HbA1c<7%) [21]. This unsatisfactory glycemic control had changed a little over the past decade. Taking the U.S., for example, only 51% of patients who had been treated with diabetes medications achieved the optimal glycemic control for the period of 2011-2014, which is about the same as the percentage for the period of 2007-2010 (52%) [22–24].

Unsatisfactory glycemic control as a result of poor DSM may cause devastating consequences including elevated rates of comorbidity, greater risks of secondary complications, more visits to urgent care settings, increased hospitalizations, and higher medical costs [25–28]. Considering the persistent poor glycemic control and the considerable biopsychosocial demands to carry out DSM activities, there has been a call for effective approaches that could help patients deal with the daily challenges relating to diabetes, improve their adherence to the recommended diabetes management regimen, and sustain the improvements in glycemic control achieved in DSM interventions [18].

## 3. Who May Help?

As described previously, patients living with diabetes must develop a broad range of competencies that could enable them to carry out DSM activities, to solve diabetes-related social issues, and to maintain psychosocial and physical well-being [29]. Occupational therapists are known to provide activity-focused treatments and psychosocial interventions by targeting multiple levels of influence, including individual capacity, family, organization, and community factors, to facilitate DSM and psychosocial adjustments to chronic disease [29, 30]. Occupational therapists' unique role in diabetes care has been increasingly recognized for their contribution to improvement in treatment adherence, DSM abilities, and health-related quality of life [31].

## 4. Occupational Therapy (OT)

Occupational therapy (OT) is a person-centered healthcare profession that aims to enhance patients' well-being by maximizing their ability to choose, organize, and satisfactorily perform the activities, or ‘occupations' in OT term, that patients need or want to do on a daily basis [11]. The core philosophic underpinning of OT is that humans are occupational beings whose ability to involve in desired and meaningful activities is essential to their well-being and health [11]. OT treatments are informed by activity analysis where demands of desired activities are broken down at the level of individual (e.g., spiritual, affective, cognitive, and physical components), environment (e.g., cultural, institutional, physical, and social environments), and tasks (e.g., steps required to perform the desire activity) [11]. Barriers at each of these levels are then identified and addressed to develop person-centered interventions to promote activity performance [11]. Central to the focus of OT interventions is developing health-promoting habits and routines, which is a key mechanism by which health-promoting behaviors can be sustained over time [32, 33]. Despite the fact that some of the strategies used in OT are shared across disciplines, occupational therapists remain irreplaceable in diabetes health care teams because they use DSM activities as the unit of analysis and as the intervention to achieve the overarching goal of facilitating diabetes self-management[11].

## 5. Occupation-Based Treatments

### 5.1. Resilient, Empowered, Active Living with Diabetes (REAL Diabetes) [11]

REAL Diabetes is a structured, manualized one-on-one OT program for patients who struggle in carrying out DSM activities. A randomized control trial was conducted recently to investigate the effectiveness of REAL Diabetes in diabetes management. In this study, eighty-one ethnically diverse young adults (aged 22.6 ± 3.5 years; English and/or Spanish speakers) were recruited [11]. They all live in Los Angeles county, have low socioeconomic status (household income ≤ 250% of the federal poverty level) and have type 1 or type 2 diabetes ([HbA1c] = 10.8%/95mmol/mol ± 1.9%/20.8mmol/mol) [11]. Participants were then randomly allocated to the intervention group (IG) to receive biweekly OT sessions guided by the REAL Diabetes manual and to the control group (CG) to receive standardized educational materials published by the National Diabetes Education Program and biweekly follow-up phone calls for 6 months [11]. Findings of this study showed a significant improvement in IG group, as compared to CG, in glycemic control ([HbA_1c_] = -0.57%/6.2mmol/mol vs. +0.36%/3.9mmol/mol,* p* = 0.01), diabetes-related quality of life (+0.7 vs. +1.7,* p *= 0.04), and habits strength for monitoring blood glucose level (+3.9 vs. +1.7,* p *= 0.05) [11]. These results suggest that structured OT DSM interventions, such as REAL Diabetes, could contribute to improving the glycemic control as well as psychosocial outcomes among patients with diabetes.

REAL Diabetes is an individually tailored OT intervention program that centers on promoting patient autonomy and establishment of health-promoting habits and routines to manage diabetes. REAL Diabetes is grounded in an adapted, diabetes-focus Lifestyle Redesign OT framework that applies activity analysis to the DSM tasks [11]. In this program, occupational therapists delivered seven constituent content modules in accordance with patients' treatment goals in patients' homes and community settings over 6 months in a minimum of 10 hours of treatment session depending on the complexion of individuals' care needs and the progress toward their goals [11]. For patients who had identified social support as a challenge, their family members were encouraged to participate in diabetes-related education workshops [11]. Moreover, a multidisciplinary care team composed of an endocrinologist and a social worker was formed for consultations regarding medical and social issues outside the scope of OT practice [11].

The intervention consists of seven content modules [11]: (1) assessment and goal setting; (2) basic self-management knowledge and skills; (3) self-advocacy in health care and community settings; (4) establishment and maintenance of health-promoting habits and routines; (5) seeking and receiving social support; (6) enhancing emotional well-being; (7) self-reflection and strategies to maintain long-term health. After completing the initial evaluation and establishment of goals in module 1, occupational therapists personalized the interventions by using content from the remaining modules that are relevant to patients' individual goals and are in line with their personal factors, including their readiness to change, personal preferences, and their prescribed diabetes management regimen [11]. REAL Diabetes is therefore a menu of possible treatment activities that can be selected to meet the needs of individual patient, rather than a fixed curriculum that every participant need to complete. In this way a person-centered intervention program was provided to address unique needs of each patient.

## 6. OT Psychosocial Interventions

Several studies have presented a viewpoint that psychosocial support is essential to dealing with the daily challenges of diabetes [17, 34]. For many patients, living with diabetes is a complex, lifelong process where the psychological consequences of continuous DSM activities and pressures from family, friends, and health care professionals can generate stress and burnout in daily life [35, 36]. In addition, patients' experiences of their body's reaction to episodes of low or high blood glucose levels could result in a fear of hypoglycemia and a loss of confidence in their bodily functions [37–41]. Individuals with diabetes hence must develop a broad range of competencies that could support them to deal with diabetes-related social issues, to redesign lifestyle and daily routines, and to maintain psychosocial and physical well-being [42–45]. Scientific evidence has shown that occupational therapists are competent to provide psychosocial interventions that target multiple levels of influence to facilitate psychosocial adjustments to diabetes and thus improve patients' ability in DSM [30, 42–45].

A pilot study has proved the efficacy of a structured peer-led group program supervised indirectly by an occupational therapist in improving adherence to the diabetes management regimen among 16 Mexican-American older adults aged 60-85 [46]. All participants have type 2 diabetes and lack motivation to establish health-promoting habits [46]. After two months, these participants saw improved glycemic control and improved self-perception of being prepared to manage diabetes. HbA1c test results were significant at the* p *< 0.05 level between pretest and 2-month posttest with a stabilizing effect found at the 6-month posttest [46]. Furthermore, changes in responses on the Diabetes Self-Efficacy Scale, the Diabetes Attitude Scale, and the Diabetes Empowerment Scale were highly significant at the 2-month, 4-month, and 6-month posttests at the* p* < 0.0005 level [46].

This peer-led OT psychosocial intervention program adopted “Bridges Diabetes Support Group Manual (BDSGM)” to structure the intervention. Each BDSGM chapter used stories to teach concepts and asked introspective questions at the end of each chapter to encourage readers to reflect on their motivations for changes and self-preservation behaviors, and to refine their problem-solving and social skills, which are considered essential competences in adhering to the prescribed diabetes management regimen [46]. The intention of this manual is to counteract negative thinking and correct faulty information by exposing participants to different viewpoints, and to provide topics for shared discussion in group sessions led by peer mentors [46]. During phase one, an occupational therapist served as a peer role model for the interventions using BDSGM [46]. A train-the-trainers strategy was used to enable selected peer mentors to run subsequent mentee groups where the occupational therapist only stood by on the premises [46]. To facilitate mentees' acceptance of and adjustment to diabetes, key issues such as health care beliefs, values clarification, changing habits, developing goals, stages of adaptation, and social assertiveness were explored introspectively and were discussed in group sessions [46].

Occupational therapists have a long history of using peer support groups to address individuals' psychosocial needs and encourage behavioral changes [42–45]. The above diabetes peer support program borrows a concept from Social Learning Theory that social support can be a powerful tool for motivation for changes because vicarious learning occurs when one recognizes and imitates others' success [47–50]. Bringing patients facing similar diabetes-related challenges together and encouraging them to share lived experiences regarding emotional and practical challenges of diabetes, this peer support program could elicit hope and motivate patients to improve their DSM abilities [51–53]. In this program, patients could give and receive emotional support from peers. The interconnections of people alike could reduce the feeling of being alone in their struggle with diabetes [54]. In addition, patients could learn how others have coped with the conditions they are struggling with [53]. Participants who have successfully managed diabetes, in particular, can deliver powerful and inspiring messages to their peers [53]. Feedback from the participants is positive. They acknowledged that social support is needed at any stages of their illness and agreed that given the topical guidelines and rules of interaction to follow, peers with diabetes can provide meaningful support to each other [46].

## 7. Pediatric OT DSM Interventions

Occupational therapists are able to support patients with childhood diabetes to develop and improve DSM skills [55]. Similar to OT DSM programs targeting adult populations, adolescent patients in pediatric DSM programs participate in individual OT sessions to learn basic DSM knowledge and skills [56–63]. What makes the pediatric programs unique is that parents are often included as indispensable part of the intervention. The goal is to adjust parental involvement in DSM to promote patients' independence in DSM and to improve patients' adherence to their diabetes management regimen [56–63]. For instance, the parent-child group interventions aim to improve parents' perceptions of their children's ability to effectively manage diabetes by providing education about the developmental aspects of DSM in adolescence [60, 64]. Occupational therapists also initiate parent-child negotiation regarding proper parental involvement in DSM and encourage renegotiation of DSM responsibilities between parents and their children during adolescence [64]. To further promote adolescent patients' independence and autonomy in DSM, occupational therapists use a variety of creative ways, such as using formal behavioral contracts for parents to reduce nagging [57] and fortnightly reminders to parents to provide positive affirmations to their children [59].

Additionally, technology is used to deliver important parts of the intervention to support the development of DSM skills of teenagers with diabetes [56–65]. For instance, in some OT programs, a cell phone meter, monitored by an occupational therapist, for blood glucose values was provided to adolescent patients [56, 57]. Parents were also given a link to access and monitor children's blood glucose data and were encouraged to cease their cues for self-management, which could also promote children's independence in DSM [56, 57]. Post-treatment outcome surveys suggested that most of the adolescent patients felt more independent in DSM and agreed that the cell-phone-based blood sugar systems had helped them establish DSM habits [57]. Occupational therapists also use online platform to promote the development of DSM skills among adolescents with type 1 diabetes [65]. Patients are encouraged to involve in the online discussion forums, trouble-shoot diabetes-related challenges, and review multimedia stories focusing on common barriers to DSM [65]. Treatment outcomes suggested a significant improvement in DSM (d = 0.30; p = 0.02), and an important improvement in HbA1c (d = -0.28;* p* = 0.27) in intervention group, as compared to control group [65].

## 8. OT Practice Challenges

Despite the fact that occupational therapists have a long history of working with a variety of populations with chronic diseases, including diabetes, most of the OT interventions are directed to the disabling consequence of diabetes, such as upper limb pain, poor vision, amputation, and depression, rather than to the secondary prevention of diabetes to address challenges of DSM [46]. This is because interventions for disabilities following uncontrolled diabetes are reimbursed while DSM treatments to prevent diabetes complications may be not [46]. Although the reimbursement systems are not yet in place in many countries, occupational therapists still have the responsibility and play a significant role in facilitating patients to manage diabetes and to continually live the life they want to live [32, 46]. Furthermore, the current secondary OT prevention services may require further refinements in delivery modality in order to reach wider diabetic populations, especially those with poor access to health care [11].

## 9. Conclusion

Against the backdrop of a global rise in the incidence of diabetes and poor adherence to the prescribed diabetes management regimen, it is urgent to refine current DSM interventions. Occupational therapists can bring values to the diabetes care team by evaluating multiple levels of influence that interact to influence patients' DSM abilities, and addressing barriers at each of these levels to improve glycemic control and enhance well-being. Occupational therapists can also support patients to develop highly complex competences and skills to self-manage diabetes and can help with planning activities of daily living in a systematic way for optimum health outcomes. Scientific evidence supports that OT interventions can improve the quality of life and adherence in people with diabetes. Therefore, it is suggested that OT interventions should be continued and built on to help address the increasing needs of populations with diabetes.

## Data Availability

The datasets used and analysed during the current study are available from the corresponding author on reasonable request.

## Abbreviations

DSM: Diabetes self-management
OT: Occupational therapy
HbAlc: Hemoglobin A1c.
